# Human immunodeficiency virus epidemic scenery among brazilian women: a spatial analysis study

**DOI:** 10.1186/s12905-023-02616-5

**Published:** 2023-09-01

**Authors:** Ana Luisa Lemos Bezerra, Paula Regina Barbosa de Almeida, Renata Karina Reis, Glenda Roberta Oliveira Naiff Ferreira, Fabianne de Jesus Dias de Sousa, Elucir Gir, Eliã Pinheiro Botelho

**Affiliations:** 1 Nursing Graduate Program, Universidade Federal do Pará, Rua Augusto Correia, 01 – Setor Saúde, Guamá – Belém, Pará, 66075-110 Brazil; 2 Escola de Enfermagem de Ribeirão Preto. Graduate Program in Fundamental Nursing, Universidade de São Paulo, Av.Bandeirantes, Ribeirão Preto, 3900, 14040-902 SP Brazil

**Keywords:** Women, HIV, Spatial analysis, Brazil

## Abstract

**Background:**

Approximately 37.7 million people worldwide are infected with human immunodeficiency virus (HIV). Although HIV detection among women, they still representing 53% of population living with the virus. Spatial analysis techniques are powerful tools for combating HIV allowing the association of the phenomenon with socioeconomic and political factors. Therefore, the main goal of this study was to spatially analyze HIV prevalence among Brazilian women from 2007 to 2020.

**Methods:**

ecological study was conducted using secondary databases of the Notifiable Diseases Information System (SINAN) for HIV and Acquired Immunodeficiency Syndrom (AIDS) in Brazilian women 15 years old and over. Age-adjusted HIV/AIDS incidence rates were analyzed using spatial distribution, autocorrelation, and spatiotemporal risk analysis techniques.

**Results:**

During the study period, 119,890 cases of HIV/AIDS were reported among Brazilian women. The southeastern region had a higher age-adjusted HIV/AIDS incidence than other Brazilian regions. Hotspot HIV/AIDS incidence rates decreased in all Brazil. Piauí, Paraná, and Minas Gerais were the only states with an increased number of cold spots. Previous spatiotemporal risk zones were observed in the states of São Paulo, Rio Grande do Sul, and Rio de Janeiro. Belém was a risk zone with a later spatiotemporal risk.

**Conclusions:**

The efficiency of public policies fighting HIV has not been uniform among municipalities, although HIV/AIDS cases have decreased among Brazilian women. The social determinants of health in each municipality should be considered when local health authorities implement policies. Women empowerment should be promoted, and access to preventive, diagnostic, and treatment healthcare places should be expanded and guaranteed.

**Supplementary Information:**

The online version contains supplementary material available at 10.1186/s12905-023-02616-5.

## Background

Approximately 37.7 million people worldwide are infected with human immunodeficiency virus (HIV) with women representing 53% of this population. Even the number of newly reported HIV cases decreased by 27% between 2010 and 2019 [1]. In 2020, about 750,000 new HIV infections were reported in women with 5000 new cases reported weekly [1]. The number of new HIV reported cases in women decreased by 7.76% in Brazil from 2011 to 2021 (2011:4716 cases; 2021:4350 cases). However, the HIV epidemic has discrepant sceneries among the Brazilian regions: while the HIV detection rate increases in northern Brazil, it decreases in the southern. Among the age groups, beween 2011 and 2021 the HIV prevalence had the greater and lower decreased, respectively, among women aged 30–34 (57.62%) and those aged 60 years old and over (23.88%) (2011: 30–34 = 32.8, ≥ 60 = 6.7; 2021: 30–34 = 13.9; ≥60 = 5.1; /100,000 inhabitants) [2].

Despite this progress, acquired immunodeficiency syndrome (AIDS) remains the main cause of mortality in women aged 15 to 49 years [3]. Women have a greater disadvantage for HIV infection than men owing to biological, cultural, and socioeconomic factors [4–9]. However, public policies addressing HIV infection among women remain scarce. In Brazil, women were included in government media campaigns against HIV only in 1999 after the first cases of HIV were reported in non-sex worker women [10].

Currently, policies devoted to this population are mainly focused on preventing mother-to-child HIV transmission during prenatal care [10]. Although Brazil expanded HIV testing to the primary health network in 2012, similar to condom distribution, and included HIV diagnosis as a mandatory report in 2014 [11, 12], to eliminate HIV by 2030, as proposed by UNAIDS, considering the social determinants of health (SDH) is necessary.

SDH refers to the socioeconomic and cultural territorial particularities in which individuals live [13]. Brazil is a high social inequity country with greater regional, states and municipaplities disparities. The north and northeast regions, in which HIV epidemic increases since 1980s, have the lower human development index (HDI) of Brazil [14]. In this sense, spatial analysis has emerged as a powerful tool for revealing the higher-pressure areas of the HIV epidemic and permitting the association of territorial factors contributing to the studied problem [15]. Most previous studies on the HIV epidemic among women employing spatial analysis have mainly focused on pregnant women [16–22] or have compared women and men [23].

Only one study performed spatial modeling of HIV and HSV-2 among women in Kenya [24] and one study performed a cohort of South African women at high risk of HIV infection based on a previously developed risk-scoring algorithm [25]. In Brazil, only seven studies that considered specific states as unit analysis [26–32].

Therefore, to reveal the real HIV epidemic scenario among Brazilian women, the main goal of this study was to spatially analyze the HIV epidemic between 2007 and 2020 considering 5570 Brazilian municipalities as analysis units. Here, we analyzed HIV/AIDS age-adjusted incidence rates by employing spatial distribution and autocorrelation, and spatiotemporal risk techniques.

## Methods

### Study design and settings

This ecological study employed secondary data from the Brazilian Notifiable Diseases Information System (SINAN) and considered Brazilian municipalities for analysis. Brazil is located on the South American continent and has a territorial area of 8,516,000 km^2^. It is divided into five political regions (North, Northeast, Southeast, Midwest, and South), 26 states, one federal district, and 5,570 municipalities [see Additional file 1]. In 2022, the Brazilian population was 203,1 million of inhabitants, with women estimated to be representing 51.12% of this population [33, 34].

Regarding resources for combating HIV, many regional inequalities remain, with the southeastern and southern regions presenting the lowest coverage of the Family Health Strategy in Primary Health Care [35]. Among the indicators that assess social inequities is HDI, which varies between Brazilian states, with a higher index in the Federal District and lower indexes in the northern and northeastern regions [36].

In Brazil the HIV cases notifications are mandatory and antiretroviral therapy are dispensable for free for all people diagnosed with the virus regardless the CD4 cells count. However, HIV-specialized healthcare centers and antiretroviral therapy dispensing places are insufficient in many Brazilian states. For example, the state of Pará has only six specialized healthcare centers to attend to people living with HIV/AIDS (PLWHA) and 33 antiretroviral therapy dispensing places for its 144 municipalities [37].

### Study population, data source, and variables

The study population consisted of all newly reported cases of HIV/AIDS among Brazilian women aged 15 years and older admitted to the Notifiable Diseases Information System (SINAN) from 2007 to 2020. We only included notifications from Brazilian home addresses. All data were double checked and inconsistencies were removed. For HIV/AIDS incidence rates, the national, state, and municipal female populations were obtained from the Brazilian Institute of Geography and Statistics.

For the HIV/AIDS incidence municipality rate calculation, we considered three age groups (15–29, 30–49, and 50 years and over) and three periods (2007–2011, 2012–2016, and 2017–2020). For each age group, the total number of HIV/AIDS cases was divided by the average age of the municipal population. Additionally, to avoid the impact of the small populations of some municipalities on the calculation of the incidence rates, all rates were adjusted by age using direct methods [38]. The results were then multiplied by 100,000.

### Spatial analysis

First, the aged-adjusted HIV/AIDS incidence rates of the municipalities were analyzed using the distribution and spatial autocorrelation techniques in ArcGIS ® version 10.5 (ESRI, Redland, California, United States). Data were analyzed for sample distribution using the Shapiro–Wilk test in RStudio software (Version 1.4; RStudio, Boston, MA, USA) before running the spatial autocorrelation analysis. The principle of autocorrelation analysis is that neigbhoor municipalities share same characteristics and it is a powerful tool to evaluate public policy eficiency.

We used the Gi* statistic autocorrelation analysis because all the samples had an abnormal distribution (Table 1) [39]. Therefore, the statistical significance of the cluster was analyzed using the Getis-Ord General G. The Getis-Ord general index (W) indicates the presence of spatial autocorrelation. Only W (p < 0.05) was considered autocorrelated.

**Table 1:** Shapiro–Wilk test results for the sample distribution analyses

	Year periods		
Brazillian region	2007–2011	2012–2016	2017–2020
North Region	W = 0.68801	W = 0.79163	W = 0.67866
	*p* < 0.01	*p* < 0.01	*p* < 0.01
Northeast Region	W = 0.72331	W = 0.77923	W = 0.72208
	*p* < 0.01	*p* < 0.01	*p* < 0.01
Midwest Region	W = 0.71001	W = 0.79063	W = 0.73105
	*p* < 0.01	*p* < 0.01	*p* < 0.01
Southeast Region	W = 0.7883	W = 0.75272	W = 0.64798
	*p* < 0.01	*p* < 0.01	*p* < 0.01
South Region	W = 0.77714	W = 0.82125	W = 0.71131
	*p* < 0.01	*p* < 0.01	*p* < 0.01

We used the Local Gi* analysis to determine the clustering locations. Local Gi* measures the degree of association of weighted points in a given distance, and its results are given in confidence intervals (CI) and grouped into 90%, 95%, and 99% CI. Clustering was classified into hotspot or coldspot intervals and municipalities sharing high- or low-low incidence rates. As proving the efficiency of public policies fighting HIV among women, we expected a decrease in number of hotspots, or a contraction in their size, and an increase in number of coldspots or the expansion of the already existing ones. Of the In all G statistics, we consider the spatial relationship of municipalities sharing borders or corners. Additionally, to be considered a cluster, it must comprise at least three municipalities.

To reveal the risk zones of the HIV epidemic, spatiotemporal risk analysis was used through a discrete Poisson model in the software SatScan 9.7 (Kulldorf, Cambridge, MA, USA). This analysis is useful to see the area more affected by the HIV epidemic and at which specific time it occurred. It provides information not only about the impacted area but also permits the identification of possible sociopolitical factors associated with the studied phenomenon in the specific time period indicated in the results.

For this analysis, we used the following criteria: non-overlapping clusters with a maximum size of 50% of the exposed population and of the studied period and 999 replications. The relative risk (RR) had to be greater than 1 to be considered a risk zone or cluster with p < 0.05 [40]. All thematic maps were constructed using the Datum Horizontal SIRGAS-2000 reference system in ArcGIS ® version 10.5 (ESRI, Redland, California, United States). The cartographic bases were obtained from the Brazilian Institute of Geography and Statistics (IBGE), and all results were shown in choroleptic scales.

## Results

During the study period, 119,890 HIV/AIDS cases were reported among Brazilian women. Age-adjusted HIV/AIDS incidence rates were higher in the southeastern region for all time periods and decreased in all Brazilian regions (Table 2).

**Table 2:** HIV/AIDS age-adjusted incidence rates by five years periods for each Brazilian region

	Brazilian region				
Year periods	North	Northeast	Midwest	Southeast	South
2007–2011	4.14	10.47	3.91	23.92	16.19
2012–2016	5.45	13.42	4.32	21.96	16.95
2017–2020	3.35	7.74	2.28	11.37	8.31

**Fig. 1:**
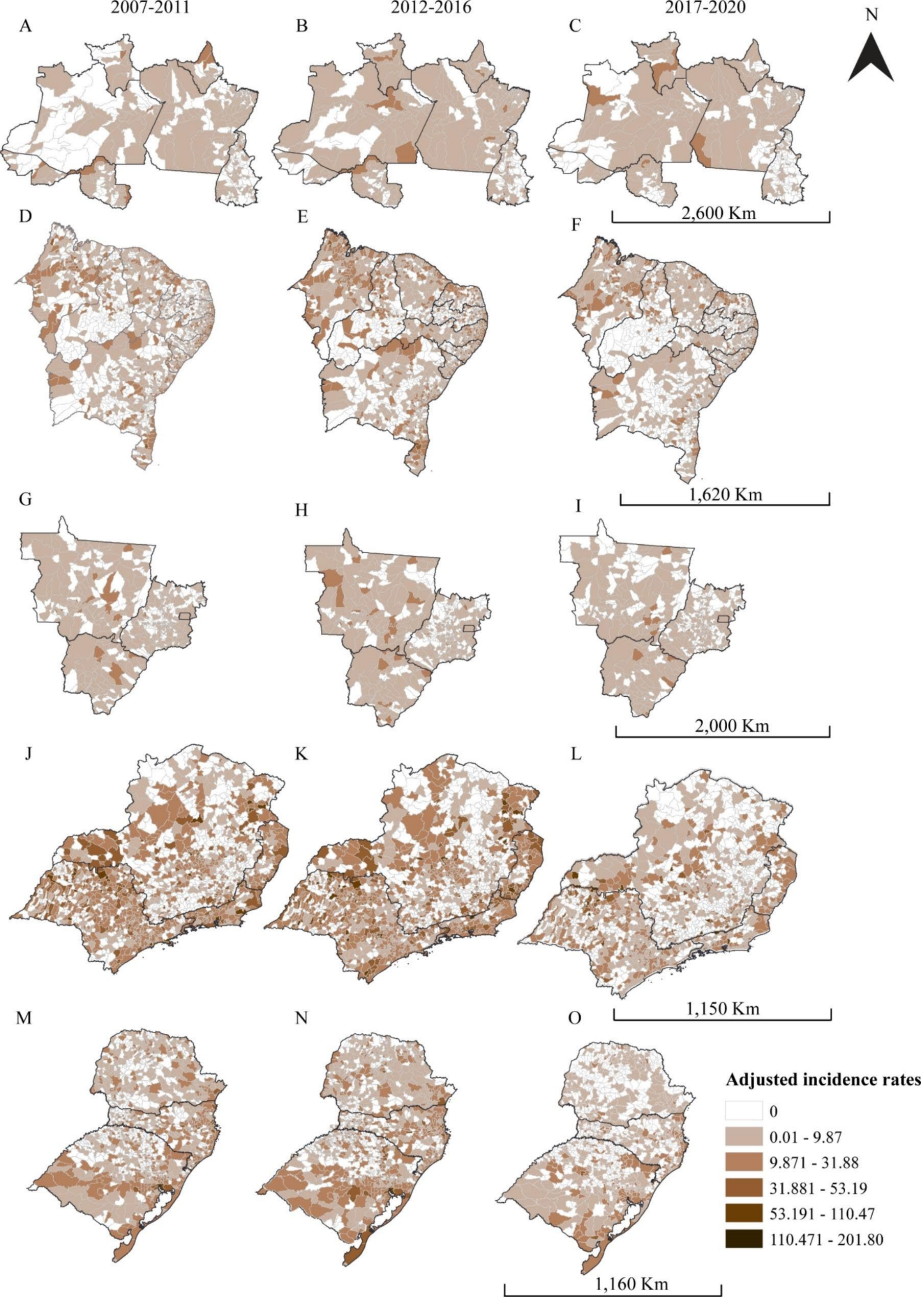
Spatial distribution of the HIV/AIDS age-standardized incidence rates among Brazilian women per five years periods for each Brazilian region: north (Fig. 1A, B and **C**), northeast (Fig. 1D, E and **F**), midwest (Fig. 1G, H and **I**), southeast (Fig. 1J, K and **L**) and south (Fig. 1 M, N and **O**)

Figure 1 illustrates the spatial distribution of the age-standardized rate of HIV/AIDS cases among women for each 5-year period, 2007–2011, 2012–2016, and 2017–2020, for all Brazilian regions: North (Fig. 1A, B, and C), Northeast (Fig. 1D, E, and F), Midwest (Fig. 1G, H, and I), Southeast (Fig. 1J, K, and L), and South (Fig. 1 M, N, and O). Some municipalities remained unchanged, while in others, the rate increased, although a territorial decrease in HIV epidemic existed over time in all Brazil. Northern, northeastern, and midwest regions had lower HIV levels.

For spatial autocorrelation, the G index was statistically significant for all regions and time periods except for the Midwest region between 2007 and 2011 (Table 3).

**Table 3:** Global Getis-Ord (G) spatiall autocorrelation results for HIV/AIDS age-adjusted incidence rates for each Brazilian region and study periods

	Year periods		
Brazilian region	2007–2011	2012–2016	2017–2020
North	Observed G: 0.01	Observed G: 0.01	Observed G: 0.01
	z-score: 2.88	z-score: 3.38	z-score: 4.63
	*p* < 0.01	*p* < 0.01	*p* < 0.01
Northeast	Observed G: 0.003	Observed G: 0.003	Observed G: 0.003
	z-score: 12.43	z-score: 12.11	z-score: 9.19
	*p* < 0.01	*p* < 0.01	*p* < 0.01
Midwest	Observed G: 0.01	Observed G: 0.01	Observed G: 0.01
	z-score: 0.36	z-score: 8.18	z-score: 5.26
	*p* = 0.71	*p* < 0.01	*p <* 0.01
Southeast	Observed G: 0.00 3	Observed G: 0.003	Observed G: 0.003
	z-score: 11.21	z-score: 9.74	z-score: 5.77
	*p* < 0.01	*p* < 0.01	*p* < 0.01
South	Observed G: 0.005	Observed G: 0.005	Observed G: 0.005
	z-score: 13.18	z-score: 10.71	z-score: 13.14
	*p* < 0.01	*p* < 0.01	*p* < 0.01

**Fig. 2:**
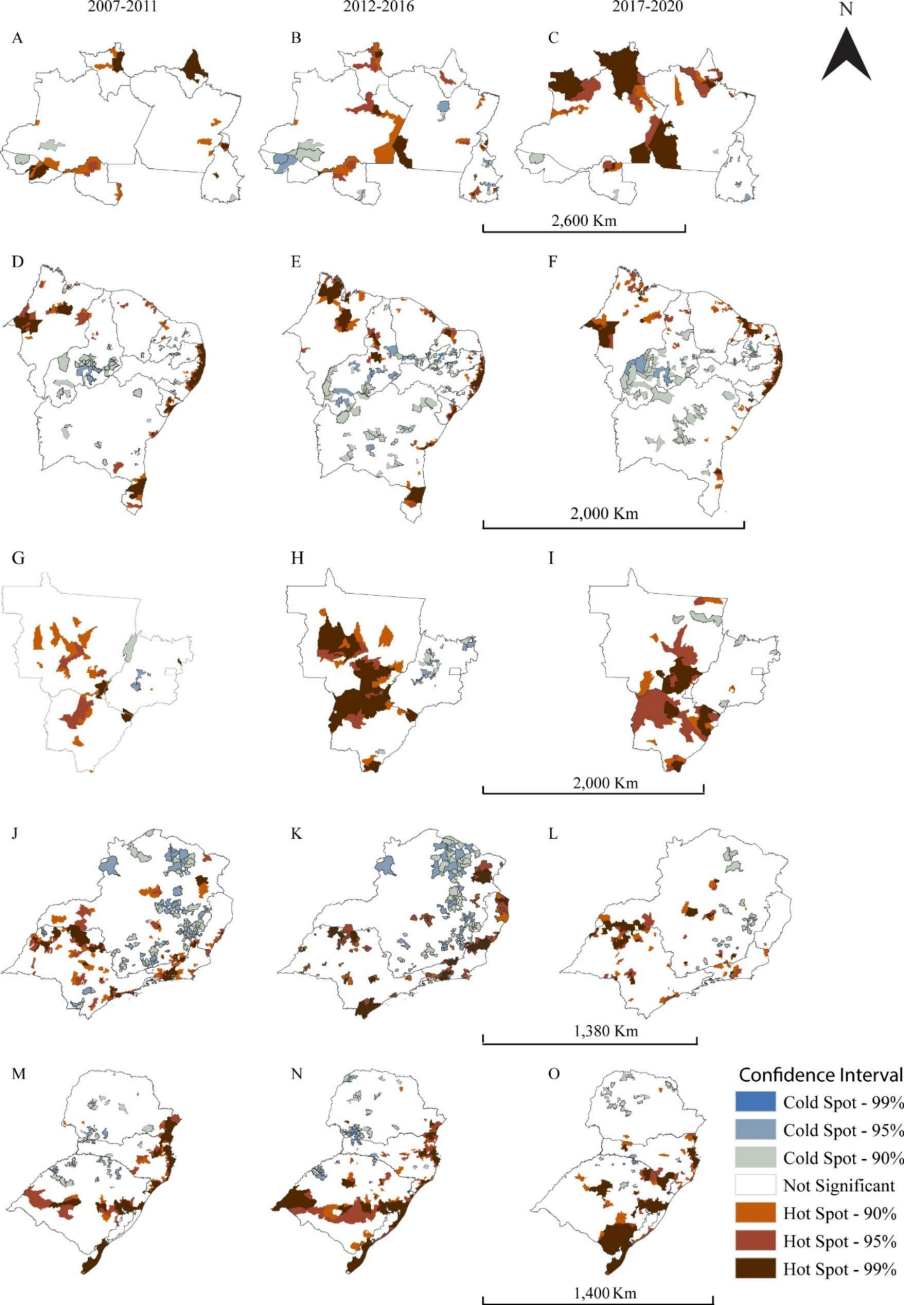
Hot- and coldspots locations of HIV/AIDS age-adjusted incidence rates among Brazilian women per five years periods for each Brazilian region: north (Fig. 2A, B and **C**), northeast (Fig. 2D, E and **F**), midwest (Fig. 2G, H and **I**), southeast (Fig. 2J, K and **L**) and south (Fig. 2 M, N and **O**)

Figure 2 shows the locations of hotspots and coldspots. We observed a reduction in hotspots in all regions over time. In the northern region, an expansion of the hotspot in the state of Rondonia into the Amazonas state, the disappearance of the Acre and northern Amapá clusters, a contraction of the cluster in Roraima were noted. The maintenance of the cluster comprised of municipalities in southeast Amazonas and southwest Pará, and an emerging new clustering in southern Amapá. (Figs. 2 A, B, and C).

The northeastern region (Fig. 2D, E, and F) also showed a decrease in hotspots; however, the states of Ceará, Rio Grande do Norte, and Alagoas increased. In addition, we noticed that most of the hotspots comprised municipalities located on the Atlantic coast, and Paraiba, Pernambuco, and Alagoas were the main states with the same hotspots during the study period. In the Midwest region (Fig. 2G, H, and I), the hotspot expanded to the west and northeast of Mato Grosso do Sul and southeast of Mato Grosso. In addition, hotspots located in southwestern Mato Grosso do Sul expanded.

In the southeastern region (Fig. 2J, K, and L), the hotspot was restricted to Minas Gerais, Rio de Janeiro, and São Paulo over time. Noticing an expansion of the cluster located in the border of São Paulo and Minas Gerais in west and southwest meridionals, respectively, is possible. The hotspots were located in the inner region of Minas Gerais, São Paulo, as well as on the Atlantic coast of São Paulo and Rio de Janeiro, which englobed other neighboring municipalities. Additionally, new small hotspot clusters emerged over time.

In the southern region (Fig. 2 M, N, and O), the hotspot clusters disappeared in Paraná and were located only in Santa Catarina and Rio Grande do Sul. Between 2017 and 2020, in Santa Catarina, three clusters were present, two of which were comprised of municipalities located on the Atlantic coast and one of the municipalities in the west meridional, together with municipalities in the northeast portion of Rio Grande do Sul.

Rio Grande do Sul had five clusters, one of them already mentioned, another cluster comprised the capital of the state, Porto Alegre, and surrounding municipalities, another located closer to the north meridional, another in the midwest portion, and one in the southeast portion bordering Uruguay. Piauí (southern), Bahia (mid portion), Bahia and Paraná (west and north meridionals) were the only states showing an increasing coldspot clusters.

**Fig. 3:**
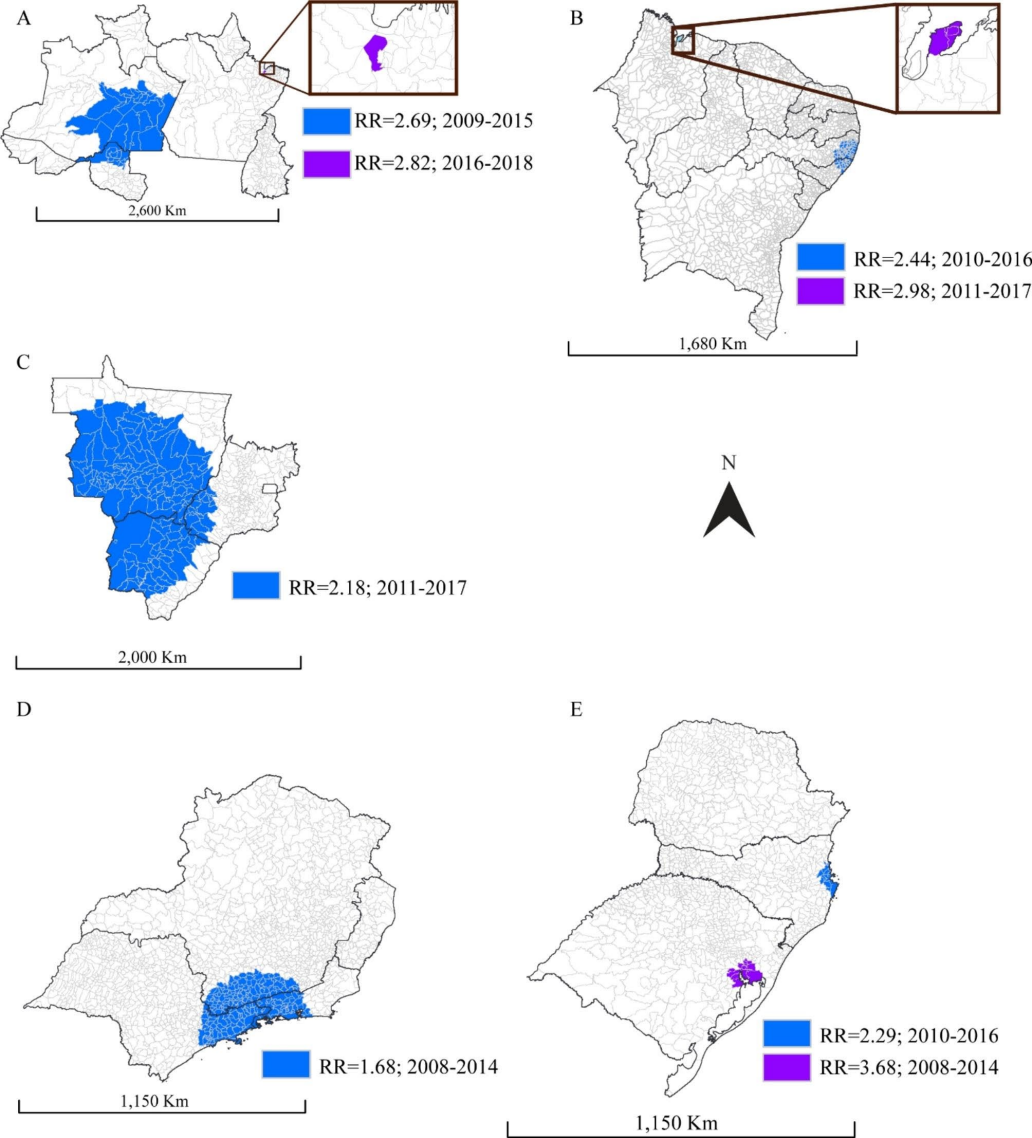
Spatio-temporal risk zone locations for HIV among Brazilian women in each Brazilian region: north (Fig. 3A), northeast (Fig. 3B), midwest (Fig. 3C), southeast (Fig. 3D) and south (Fig. 3E)

Figure 3 illustrates the HIV spatiotemporal risk zones. In the northern region, two risk zones existed, one zone being the capital of Pará, Belém (RR = 2.82, p < 0.001; 2016–2018) and the other comprising the municipalities of southern Amazonas and northern Rondônia (RR = 2.69, p < 0.001; 2009–2015) (Fig. 3A). Similarly, in the Northeast region, two risk zones were present: one composed of the municipalities of Maranhão (Paço do Lumiar, Raposa, São José de Ribamar, and São Luís) (RR = 2.98, p < 0.001; 2011–2017) and the other by the municipalities of Pernambuco and Alagoas (RR = 2.44, p < 0.001; 2010–2016) (Fig. 3B).

In the Midwest Region, the risk zone comprised the municipalities of Mato Grosso, Mato Grosso do Sul, and southwest Goiás (Fig. 3C) (RR = 2.18, p < 0.001; 2011–2017) (Fig. 3C). In the Southeast region, the risk zone comprised the municipalities of Rio de Janeiro, São Paulo, and Minas Gerais (RR = 1.68, p < 0.001; 2008–2014) (Fig. 3D), and in the southern region by Porto Alegre and neighboring municipalities (RR = 3.68, p < 0.001; 2008–2014).

## Discussion

Our results revealed the real scenario of the HIV epidemic among Brazilian women between 2007 and 2020. However, the adjusted incidence rates of HIV/AIDS decreased over time in all regions of Brazil. The HIV epidemic has decreased territorially in all regions of Brazil. Although a decrease or shrinkage of hotspots could be seen in most Brazilian states, in some states, it increased, as in Roraima, Mato Grosso do Sul, Rio Grande do Norte, Ceará, and Alagoas. The only states that exhibited an increase in coldspots were Piauí, Bahia, and Paraná. Rio Grande do Sul was the state with the greatest spatiotemporal RR, with the cluster comprising its capital and municipalities. Belém was the only risk zone with a later and shorter spatiotemporal risk.

Although there was a territorial decrease of the HIV epidemic in Brazil, in some states in the northern, mid-west, and northeastern regions, this decrease was lower. These regions have particularities that make HIV combat difficult, such as large territorial areas, low demographic density compared with other Brazilian regions, low socioeconomic conditions of their inhabitants, lower access to technologies, people living far away from healthcare centers, and low coverage of preventive and HIV treatment healthcare centers. In Mato Grosso do Sul, for example, of its 79 municipalities, only 14 healthcare centers specialize in attending to people living with HIV/AIDS [37, 41].

Sexual exploration also suggest hotspot expansion in the northern, northeastern, and midwest regions. In Northern Brazil, deforestation associated with illegal mining and construction of highways has promoted an increase in prostitution and sexually transmissible infections, logging on indigenous lands, construction of dams and agricultural projects, tourism, transit in border regions, and settlement of indigenous populations in urban areas [42]. In Pará, a study showed a high prevalence of syphilis (63.41%) among female sex workers in municipalities crossed by federal highways [43].

Hotspot expansion was noticeable in Roraima during the study period. Roraima borders Venezuela and Guyana, a region with a high quantity of illegal mining and sexual exploration, in addition to being part of the drug traffic route and receiving a large number of Venezuelan immigrants per year. The Venezuelan immigration also caused a great increase in HIV rate detection in North Santander, Colombia. North Santander bordes Venezuela and miggrant Venezuellans were responsible for 33.33% of all HIV notification in 2017 [44]. Additionally, from 2018 to 2022, illegal mining in Roraima will increase with the invasion of indigenous reserves owing to the anti-indigenous positions of the former Brazilian government [45].

Illegal drug traffic may also explain the observed expansion of the hotspot on the border of São Paulo, Minas Gerais, and in the midwest region, a passageway for trafficking drugs for all of Brazil and other countries. A study in a city of a midwest region, Goiânia, showed a high prevalence (6.61%) of HIV among women using cocaine crack, and a great percentage had transmitted HIV drug resistance [46].

Furthermore, sexual tourism can also be another factor to explain the expansion of hotspots noticed in the northeast continenat coast. These cities are highly touristic, and sexual tourism can be implied for the expansion of the HIV epidemic. A previous study showed a possibility of 6.61% HIV infection in people aged 15–49 years old for evey 1,000,000 increment in internation arrivals [47].

We expected an increase in coldspots in all Brazilian states as noticed in the interior of Piauí, mid portion of Bahia and western Paraná proving the efficiency of public policies fighting HIV among Brazilian women. However, noting only a decrease in the number of hotspot clusters suggests an absence of efficacy uniformity in public policies among municipalities. For an efficient policy, it is necessary to consider that the social determinants of health act differently during the HIV epidemic in each territory. In the municipalities of Alagoas, the risk of HIV infection varies with the coverage of primary healthcare facilities, people’s schooling levels, and social inequities [48].

The spatiotemporal risk zones identified in our study comprised periods of important public policies fighting HIV among Brazilian women, such as the Stock Network Program (2011), implementation of HIV tests during prenatal care (2012), and decentralization of the HIV test to primary healthcare places (2014) [49, 50]. Addiotionally, states and municipalities efforts on expanding specialized healthcare places to assist people living with HIV and of primary healthcalre coverage contribute to decrease HIV. Between 2013 and 2019 the Health Family Strategy expanded 11.6% and this expansion was greater among more vulnerable communities [51].

Belém was a risk zone having a delayed temporal risk (2016) compared with other zones, which is suggestive of a latency in implementing policies combating HIV by the local health authorities. A previous study employing a time series of HIV/AIDS incidence rates among adults living in Belém between 2007 and 2018 showed a peak in the HIV/AIDS incidence rate in Belém in 2016 followed by a downward trend. This peak was explained by a great campaign against HIV that year, which was followed by discontinuation [52].

Rio Grande do Sul, São Paulo, and Rio de Janeiro had previous spatio-temporal zone risks, which may have been due to the faster implementation of public policies fighting HIV. In addition, compared with southern Brazil, people living in the north, northeast, and midwest regions have lower access to healthcare services because of low socioeconomic conditions, low schooling, lower access to technologies, low coverage of medical doctors, and living far from healthcare services [53]. Furthermore, the spatiotemporal risk results showed that risk zone areas comprised the capital of states and cities, suggesting that public policies are more efficient in areas closer to the capital.

This study was limmited by the undereporting HIV/AIDS cases during the pandemic COVID-19 oubreak in 2020 due to reallocation of professionals to work in pandemic scenery. Therefore, incidence rates could be understimated.

## Conclusions

Although HIV/AIDS incidence rates decreased among Brazilian women, autocorrelation analysis showed expansion of hotspots in north, northeast and midwest regions suggesting low efficiency of public policies fighting HIV. Coldspots increased only in Piauí, Paraná, and Bahia, suggesting more efficiency-focused public policies in these states. The spatiotemporal risk zones comprised capitals and municipalities, which suggests a restricted distance influence from the capital of the policies combating HIV.

### Electronic supplementary material

Below is the link to the electronic supplementary material.

Supplementary Material 1

## Data Availability

Data are free available on DATASUS website [https://datasus.saude.gov.br/informacoes-de-saude-tabnet/].

## Abbreviations

AIDS: Acquired Immunodeficiency Syndrom
CI: Confidence Interval
HDI: Human Development Index
HIV: Human Immunodeficiency Virus
IBGE: Brazilian Institute of Geography and Statistics
PEP: Pós-exposure prophyilaxies for HIV
PHC: Primary Health Care
PLWHA: People Living With HIV/AIDS
PREP: Pré-exposure prophyilaxies for HIV
RR: Relative Risk
SDH: Social Determinants of Health
SINAN: Brazilian Notifiable Diseases Information System
