# Correction to: Human immunodeficiency virus epidemic scenery among brazilian women: a spatial analysis study

**DOI:** 10.1186/s12905-023-02655-y

**Published:** 2023-09-15

**Authors:** Ana Luisa Lemos Bezerra, Paula Regina Barbosa de Almeida, Renata Karina Reis, Glenda Roberta Oliveira Naiff Ferreira, Fabianne de Jesus Dias de Sousa, Elucir Gir, Eliã Pinheiro Botelho

**Affiliations:** 1 Nursing Graduate Program, Universidade Federal do Pará, Rua Augusto Correia, 01–Setor Saúde, Guamá– Belém, 66075-110 Pará, Brazil; 2 Escola de Enfermagem de Ribeirão Preto. Graduate Program in Fundamental Nursing, Universidade de São Paulo, Av.Bandeirantes, 14040-902 Ribeirão Preto, SP Brazil

Following publication of the original article [1], in this article the author name “Fabianne de Jesus Dias de Sousa” was incorrectly written as “Fabiana de Jesus Dias de Sousa”.

The original article has been corrected.
