# Supplementary figures and images for: Human immunodeficiency virus epidemic scenery among brazilian women: a spatial analysis study
Source: BMC Womens Health. 2023 Sep 1;23:463. doi: 10.1186/s12905-023-02616-5 (PMC10474736; doi:10.1186/s12905-023-02616-5)

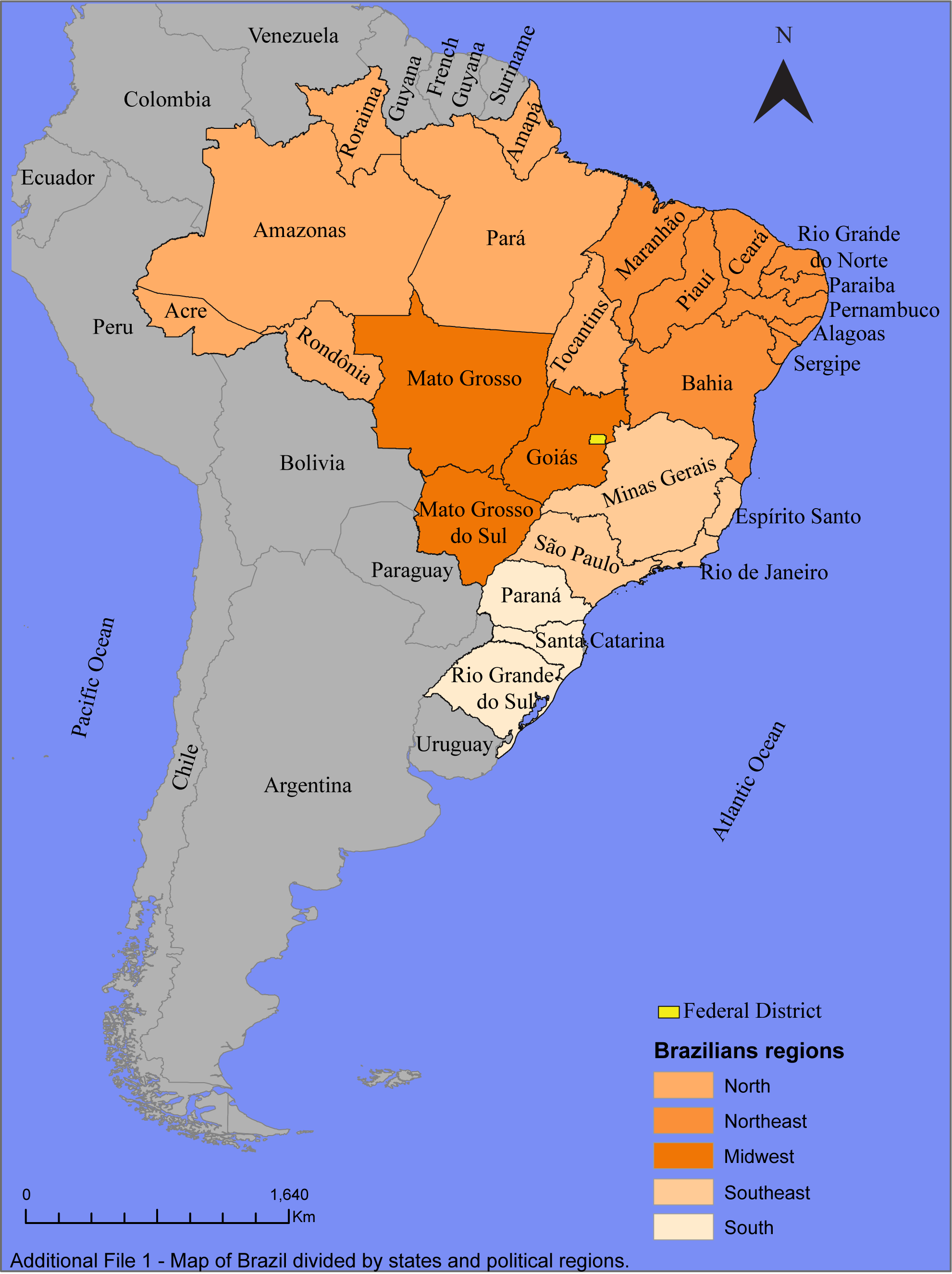

Supplement: Supplementary file 1 — Supplementary Material 1 [file 12905_2023_2616_MOESM1_ESM.tif]
